# *In vitro* antioxidant, anticholinesterase, and antiproliferative activities of methanol extracts of *Crateva religiosa* bark

**DOI:** 10.7150/jca.94492

**Published:** 2025-04-28

**Authors:** Md Sifat Hossain, Md Abdul Majed Patwary, Sharmin Akther Rupa, Mohsin Kazi, Ashok Kumar, Khalid M Alghamdi, Mohammed Mahbubul Matin, Md. Rezaur Rahman

**Affiliations:** 1 Department of Pharmacy, Comilla University, Cumilla-3506, Bangladesh.; 2 Institute for Biomedical Science, Georgia State University, Atlanta, Georgia.; 3 Department of Chemistry, Comilla University, Cumilla-3506, Bangladesh.; 4 Department of Pharmaceutics, College of Pharmacy, PO BOX 2457, King Saud University, Riyadh, 11451, Saudi Arabia.; 5 Vitiligo Research Chair, Department of Dermatology, College of Medicine, King Saud University, Riyadh 11451, Saudi Arabia.; 6 Department of Chemistry, University of Chittagong, Chittagong, 4331, Bangladesh.; 7 Department of Chemical Engineering and Energy Sustainability, Faculty of Engineering, University Malaysia Sarawak, Malaysia.

**Keywords:** *Crateva religiose*, antioxidant, anticholinesterase, and antiproliferative

## Abstract

**Background:** *C. religiosa* has traditionally been applied to treat heart disease, chronic weight loss, improved digestion, memory loss, convulsion and epilepsy, psychological problems, and neurologic pains.

**Purpose:** Prior studies have already elucidated the potential therapeutic effects of C. religiosa. However, in this work, the bark extract of *C. religiosa* was studied systematically to investigate its antioxidant, anticholinesterase, and antiproliferative activities, focusing on potential applications in treating Alzheimer's disease (AD) and cancer.

**Study Design and Methods:** The dried coarse powder of *C. religiosa* bark was cold-extracted in methanol and evaporated to dryness. It was then successively fractionated into petroleum ether (PEF), dichloromethane (DMF), and ethyl acetate (EEF) fractions. The Folin-Ciocalteu reagent and AlCl_3_ approaches were utilized to evaluate the total phenol and flavonoid contents, respectively, and the DPPH (2,2-diphenyl-1-picrylhydrazyl) and phosphomolybdenum assays were employed to determine the antioxidant activity of each fraction. The DMF was tested against acetylcholinesterase (AChE) and butyrylcholinesterase (BChE) by employing the Ellman method, while all fractions were tested for antiproliferative activity against HepG2 and A549 cell lines by MTT assay.

**Results:** The DMF displayed the highest phenolic content (124.8 ± 17.5 mg gallic acid equivalent/g of dry extract) and flavonoid content (211.1 ± 4.8 mg quercetin equivalent/g of dry extract). In the phosphomolybdenum assay, its antioxidant IC50 value was 25 ± 1 µg/mL. Additionally, the DMF fraction exhibited significant inhibition activity against AChE and BChE, with IC50 values of 455 ± 1 and 450 ± 1 μg/mL, respectively. In terms of anti-proliferative activity, the DMF exhibited an IC50 value of 29.2 µM against the HepG2 cell line, while the EAF showed IC50 values of 24.7 µM in the A549 cell line.

**Conclusion:** The potent activity of *C. religiosa* as an antioxidant, along with its significant inhibition of AChE and BChE, positions it as a promising candidate for Alzheimer's disease treatment. Furthermore, its robust inhibition of human liver and lung cancer cells suggests its potential as a therapeutic agent against cancer.

## 1. Introduction

Despite steady advances in the development of effective anticancer medications, cancer continues to be a fatal and destructive illness. With an estimated 9.6 million deaths and 18.1 million new cases in 2018, cancer is the second leading cause of mortality worldwide, behind only cardiovascular illnesses 1. In 2010, due to cancer, a surprising total annual economic burden was estimated to be approximately USD 1.16 trillion 2. The current standard of care for cancer treatment includes conventional methods, such as surgery, chemotherapy, and radiation therapy, and CAM approaches, including the use of natural products. More than 200 anticancer medications have been licensed worldwide in the last 60 years, and almost half of them are derived from natural sources 3. Several promising, innovative natural compounds have undergone preclinical and clinical testing for anticancer activity. However, clinical trials for the vast majority of these candidate compounds have been terminated or abandoned 3. Nearly 60 % and 80 % of the global population, especially in poor countries, rely on herbal medicine 4, demonstrating the widespread use of herbal medicine by indigenous communities around the world for the treatment of a wide range of pathological conditions. However, the presence of antineoplastic activity in more than 3000 different herbal plants has been established 5, and thirty natural compounds originating from plants have been tested as anticancer medications in clinical trials 6. Several plant-based natural compounds, including camptothecin, etoposide phosphate, paclitaxel, vinblastine, podophyllotoxin, topotecan, and homo-harringtonine, have been successfully used as anticancer chemotherapeutic drugs 7. Moreover, plant-derived anticancer molecules have fewer toxic effects on normal cells than their synthetic counterparts via alternative cell death-initiating mechanisms 8. It has been reported that mixtures of phytochemicals exist in a balanced diet and show synergistic activity, preceding increased anticancer effects and health advantages. This cannot be achieved by using the consumption of a single functioning compound 9, 10. As a result, there has been a surge in recent years in the study of herbal plants as potential resources of anticancer medications, with the hope of using them as natural therapeutic alternatives to conventional cancer treatments 6.

Alzheimer's disease (AD) is a complex multifactorial neurodegenerative disorder that is a leading cause of dementia and strongly affects brain function. It leads to severe memory loss and weakened language and judgment ability among older people. The disease can also affect younger individuals. According to an epidemiological survey, approximately 7-10 % of the population over 65 and 50-60 % of those over 85 are affected by AD, totalling almost 35 million people worldwide 11, 12. AD can be identified by the development of senile plaques, which are composed of neurofibrillary tangles (NFTs), amyloid beta protein, and a deterioration of both cortical and cholinergic neurons 13,14. The cognitive problem in AD patients occurs by the defeat of the neurotransmitter acetylcholine, which controls the function of cholinergic neurotransmission 15. In the cerebral cortex, the AChE enzyme is liable for the failure of acetylcholine within synapses. Therefore, AChE inhibitors can be commonly used to treat AD 16, 17.

Numerous metabolic processes and a wide variation of external factors are liable for forming free radicals within the body^18^. The abundant accumulation of reactive oxygen species (ROS) in the body is responsible for several chronic diseases, including AD, which is caused by free radical-induced oxidative stress 19. By generating antioxidants to destroy excess oxidants, the antioxidative defense scheme works to prevent the injurious influences of oxidative stress 20, 21. At present, several AChE inhibitors are used to treat AD, which can cause severe adverse effects 22, 23. Moreover, synthetic antioxidants are also applied in many cases, which have adverse effects, including liver damage and cancer 24. On the other hand, natural plants contain a wide and largely unexplored source of compounds for drug discovery, including the advancement of novel antioxidants and cholinesterase inhibitors.

*Crateva religiosa (C. religiosa),* a member of the Capparaceae family, is a medium-sized deciduous tree also known as Varuna or the Garlic Pear Plant. Several such species are widely distributed in Australia, Bangladesh, Cambodia, China, Japan, Indonesia, India, Myanmar, Malaysia, New Guinea, Sri Lanka, Thailand, the Philippines, the Solomon Islands, and Vietnam 25. There are several reports on the bioactive 26 and pharmacological effect of *C. religiosa* as traditional medicine, used by local practitioners to treat memory loss, psychological problems, neurologic pains, convulsion and epilepsy, heart disease, chronic weight loss, improved digestion, and swelling as well as burning sensations in the soles of feet 25, 27-28, 29. There is a strong connection between some of these disorders and AD 30. Parveen and co-workers recently reported on the natural compounds extracted from *C. religiosa* leaves and studied their structure clarification, DNA binding, as well as molecular docking 31. Sahoo et al. demonstrated the antimycotic activity of C. religiosa hook and forst against designated fungal pathogens. In contrast, Bani and coworkers examined the inhibition of T lymphocyte activity using lupeol extracted from C. religiosa 32, 33. Meenaloshini et al. studied the *in vitro* anticancer effectiveness of hydroalcoholic on human ovarian cancer cells extracted from *C. religiosa* G. Forst. bark 34, which efficiently suggested the vigorous study of this plant. Here, we report a systematic investigation of the antioxidant, anticholinesterase, and antiproliferative activities of *C. religiosa* bark extract to evaluate its activity for the treatment of AD as well as lung and liver cancer cells.

## 2. Materials and Method

### 2.1. Collection of plant materials

*C. religiosa* bark was gathered from Chauddagram, Cumilla, Bangladesh, in October 2022 and classified by Mr. Khandakar Kamrul Islam, a senior scientific officer, and the National Herbarium, Dhaka, Bangladesh, with a voucher specimen (DACB-86525). The bark was then cleaned with clean water to eliminate foul materials, after which, for several days, the plants were dried in the shade. The dried bark was then crushed into a coarse powder employing a crushing machine, and the resulting bark powder was stored at room temperature (RT).

### 2.2. Preparation of extract

A total of 800 g of dried powder bark was taken in an amber color extraction bottle (size: 2.5 L), and the materials were properly soaked in CH_3_OH. The bottle was sealed properly and preserved with occasional shaking for a week. The extracts were initially filtered through cotton and then again by filter paper. With a rotary evaporator at 50 °C under reduced pressure, it was then kept to get a concentrated solution. Finally, it was fractionated by petroleum ether (PEF), dichloromethane (DMF), and ethyl acetate (EAF) to gain the PEF (15.75 gm), DMF (6.23 g), and EAF (10.41 gm), as demonstrated in Flowchart S1 (Supplementary file).

### 2.3. Chemicals

1,1-Diphenyl-2-picrylhydrazyl (DPPH), phosphate buffer, gallic acid (GA), catechin (CA), ascorbic acid (AA), AlCl_3_, potassium acetate, quercetin (QU), DMSO, and methanol were obtained from Sigma Chemical Co. (St. Louis, MO, USA). Folin-Ciocalteu phenol reagent and Na_2_CO_3_ were gained from Merck (Darmstadt, Germany). MTT (3-(4, 5-dimethyl-thiazol-2-yl)-2, 5-diphenyltetrazolium bromide), DMEM, fetal bovine serum (FBS), and penicillin/streptomycin were found from Gibco (Invitrogen, Eugene, OR, USA). Furthermore, 96-well plates, T-75, T-25 flasks, pipette tips, and serological pipettes were purchased from Corning, USA.

### 2.4. Determination of DPPH free radical scavenging action

The antioxidant capacity of the diverse fractions of *C. religiosa* was studied via the colorimetric technique using the DPPH reagent, where AA was employed as a standard 35. The stock solution was serially diluted to achieve 200, 100, 50, 25, 12.5, and 6.25 μg/ml. Each test tube contained 2 ml of each concentration, and the media was properly marked. Three millilitres of 0.004 % DPPH solution were mixed in each test tube to a final volume of 5 ml, and the mixture was incubated at RT for 30 min in the dark. At 517 nm, the absorbance was subsequently evaluated against a blank. Without any sample, the control was set as previously mentioned. The free radical scavenging action was determined based on the percentage (%) of DPPH radicals scavenged utilizing the equation:

% inhibition = (1- 
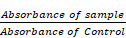
) × 100

### 2.5. Phospho-molybdenum assay

For the estimation of total antioxidant activity, the Prieto et al. method was employed 36. In this test, 0.1 ml of the sample at diverse concentrations (6.25-200 μg/ml) was reacted with 1 ml of reagent containing 0.6 M H_2_SO_4_, 4 mM ammonium molybdate, and 28 mM sodium phosphate. In a water bath, the test tubes were incubated at 95 °C for 90 min. The absorbance of the samples was subsequently estimated at 765 nm after cooling to RT, where AA was used as the control. The antioxidant activity was measured by employing the equation:

Antioxidant activity, % = (1- 
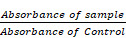
) × 100

### 2.6. Determination of total phenolics

The total phenolic content of the diverse fractions of *C. religiosa* was measured using the technique illustrated by Veliogluet in 1998, relating Folin-Ciocalteu reagent (FCR) as an oxidizing agent and catechin as a standard 37. A total of 0.5 mL of each serially diluted plant extract and standard of diverse concentration of solutions was reserved in the test tubes. In a test tube, 3 ml of diluted FCR solution with 4 ml of 7 % sodium carbonate was added and mixed well. To complete the reaction, the test tubes were incubated at RT for 30 min. Employing a spectrophotometer, the absorbance of the solution was subsequently estimated at 765 nm against a blank. The total phenolic content was measured from the calibration curve, which was developed by formulating a GA solution. The total phenolic content was stated in mg of gallic acid equivalents (GAE) per g of the dried sample.

### 2.7. Determination of total flavonoids

The total flavonoid content of the diverse fractions of *C. religiosa* was estimated by the AlCl_3_ colorimetric technique, where quercetin was used as a standard 38. In test tubes, two milliliters of each diluted plant extract and standard solutions of diverse concentrations were mixed. 2 ml of AlCl_3_ solution, 1 M potassium acetate (0.20 ml), and distilled H_2_O (5 ml) were further added. Then, to complete the reaction, the mixture was incubated for 60 min at RT. Using a spectrophotometer, the absorbance of the mixture was subsequently measured against a blank at 415 nm. The total flavonoid content was calculated from the calibration curve, which was developed by preparing a quercetin solution. The total flavonoid content was stated in mg of quercetin equivalents (QE) per g of the dried sample.

### 2.8. Determination of cholinesterase inhibitory activity

Since DMF had the best antioxidant capacity in the antioxidant tests, it was selected for the *in vitro* AChE inhibitory activity assay at 25, 50, 100, 200, and 500 μg/ml. With acetylthiocholine iodide as a substrate, the AChE inhibitory capacity of DMF was determined following the colorimetric technique 39. By a homogenizer with five volumes of ice-cold homogenization buffer, rat brains were homogenized (10 mM Tris-HCl (pH 7.2), which included 50 mM MgCl_2_, 1 M NaCl, and 1 % Triton X-100) and centrifuged at 4 °C for 30 min at 10000 × g, which was employed as the enzyme resource. As an enzyme source, the supernatant was employed after centrifugation. The protein concentration was confirmed by applying a bicinchoninic acid kit (Sigma Co., St. Louis, MO, USA) in the presence of bovine serum albumin (BSA), which served as a protein standard. DMF (500 μL), used as a standard, was combined with an enzyme (500 μL) and incubated at body temperature for 15 min. After mixing Ellman's reagent in 50 mM sodium phosphate buffer (pH = 8), the absorbance of the reaction was measured at 405 nm thoroughly. To confirm that the reaction ensued linearly, the readings were repeated 5 times at 2-minute intervals. Donepezil was applied as a positive control in this test. The % inhibition of AChE capacity was determined utilizing the following formula:

% inhibition of AChE capacity = 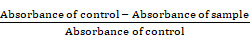
× 100

During this experiment, acetylthiocholine iodide was substituted with butyrylthiocholine iodide, and BChE inhibition was determined, as illustrated earlier, by altering the volume of the enzyme solution to 50 μl. Galantamine was applied as a positive control in this test. The % inhibition of BChE capacity was obtained by applying the same formula for AChE activity.

### 2.9. Cell lines and culture conditions

Two human cancer cell lines were employed in this research, namely, adenocarcinomic human alveolar basal epithelial cells (A549) and hepatocellular carcinoma (HepG2) cells. For the cytotoxicity comparison study, noncancerous alveolar basal epithelial cells and normal hepatocyte cell lines were used. A549 and HepG2 cells were cultured in DMEM enhanced with 10 % FBS and 1 % penicillin/streptomycin at 37 °C, 5 % CO_2_, and 95 % air in a 75 cm^2^ tissue culture flask.

### 2.10. MTT assay

The alteration of MTT (yellow) to formazan crystals (purple) by mitochondrial dehydrogenase of viable cell enzymes was applied to evaluate the degree of cellular cytotoxicity employed by Com1, DMF, PEE, and EAF, as described by Kazi et al. 40 and improved by Kumar et al. 41. Concisely, in 96-well plates (2 × 10^3^ cells/well), cancer cells were placed by keeping in a complete cell growth medium and incubated at 37 °C for a day under a humidified atmosphere of 5 % CO_2_. Then, the cell medium was put back to the cell growth medium in the presence of 5 % FBS (5 % medium) and different concentrations of Com1, DMF, PEE, and EAF, such as 1.95, 3.9, 7.8, 15.6, 31.25, 62.5, 125 and 250 µg/ml, with the control (no further treatment). After 72 hours of incubation, the medium in all test and control wells was replaced with 100 µL/well MTT solution (0.5 mg/mL in PBS), and the cells were incubated at 37 °C for an additional 3 h. Afterwards, the MTT solution was replaced with 100 µL of isopropanol/well to disperse the formazan (purple) crystals that had been produced at the bottom of the wells, and the mixture was shaken at RT for at least 2 h. Later, with a Bio-Tek microplate reader (ELX 800; Bio-Tek Instruments, Winooski, VT, USA), the color intensity at 549 nm in the wells was computed. The outcomes were examined in triplicate, and the % of viable cells was calculated. The data obtained are represented as % of viable cells in the test wells in comparison to the control group. To get the cell viability, the subsequent equation was applied 41:

% cell viability = [
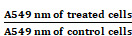
] × 100

### 2.11. Statistical analysis

The data were studied using Microsoft Excel 2016 (Roselle, IL, USA), IBM SPSS Statistics 26, and the Statistical Package for Windows version 17.0 for the Social Sciences (SPSS; IL, USA). All tests were done in triplicate, and most of the obtained data are denoted as the mean ± standard deviation.

One-way ANOVA was employed to compare the mean values of quantitative variables across the categorical variable. A post hoc Tukey HSD test was applied to validate whether there were any significant changes between the diverse concentrations of the treated groups and the control. A p-value of < 0.05 was measured as statistically important. Data were studied employing GraphPad Prism 4.0 (GraphPad Software, San Diego, CA, USA), as shown in **Tables S1 and S2.**

## 3. Results

### 3.1. DPPH free radical scavenging assay

**Figure 1:**
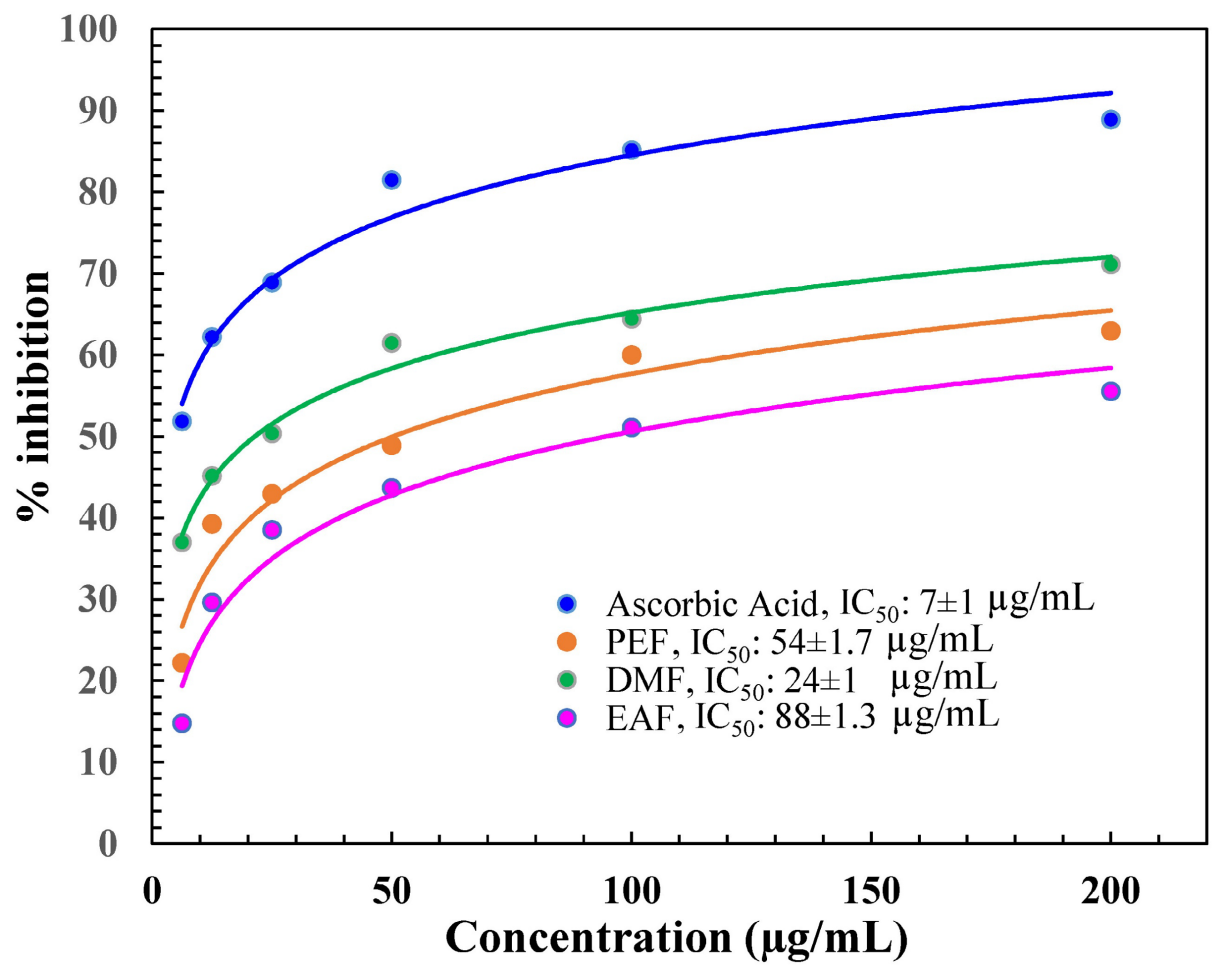
DPPH free radical scavenging activity of DMF, PEF, and EAF.

The antioxidant capacity of the diverse fractions of *C. religiosa* was expressed as the IC_50_ value. The IC_50_ is the concentration of the fraction essential to result in 50 % greater antioxidant activity than that of the control (AA). The outcomes represented that the IC_50_ value of AA was (7 ± 1) µg/mL as displayed in Figure 1. Among the tested fractions, the IC_50_ values were in the following order: DMF (24 ± 1), PEF (54 ± 1.7), and EAF (88 ± 1.3) µg/mL, which were determined from the % of inhibition value (Figure 1).

### 3.2. Phosphomolybdenum assay

**Figure 2:**
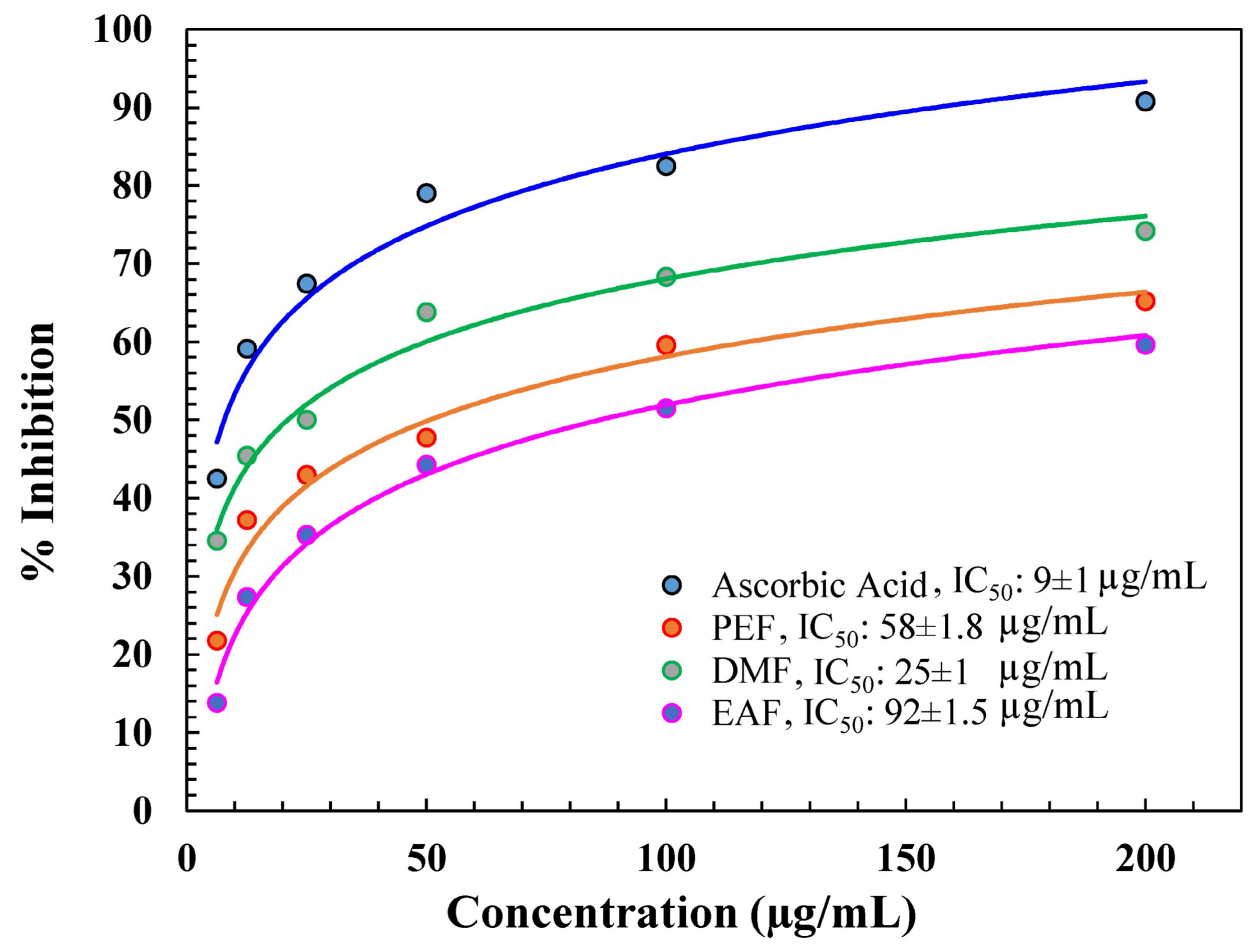
% inhibition of different fractions in the *phosphomolybdenum assay*.

In this assay, the decrease of Mo(VI) to Mo(V) by a reductant was determined by the development of a green phosphate-Mo(V) complex. Among the fractions, DMF showed the best total antioxidant activity, i.e., the IC_50_ value was 25 µg/ml, as shown in Figure 2. Other fractions did not show good IC_50_ values or molybdenum ion percentage reductions (Figure 2) compared with AA (9 µg/ml).

### 3.3. Determination of total phenolics

**Figure 3:**
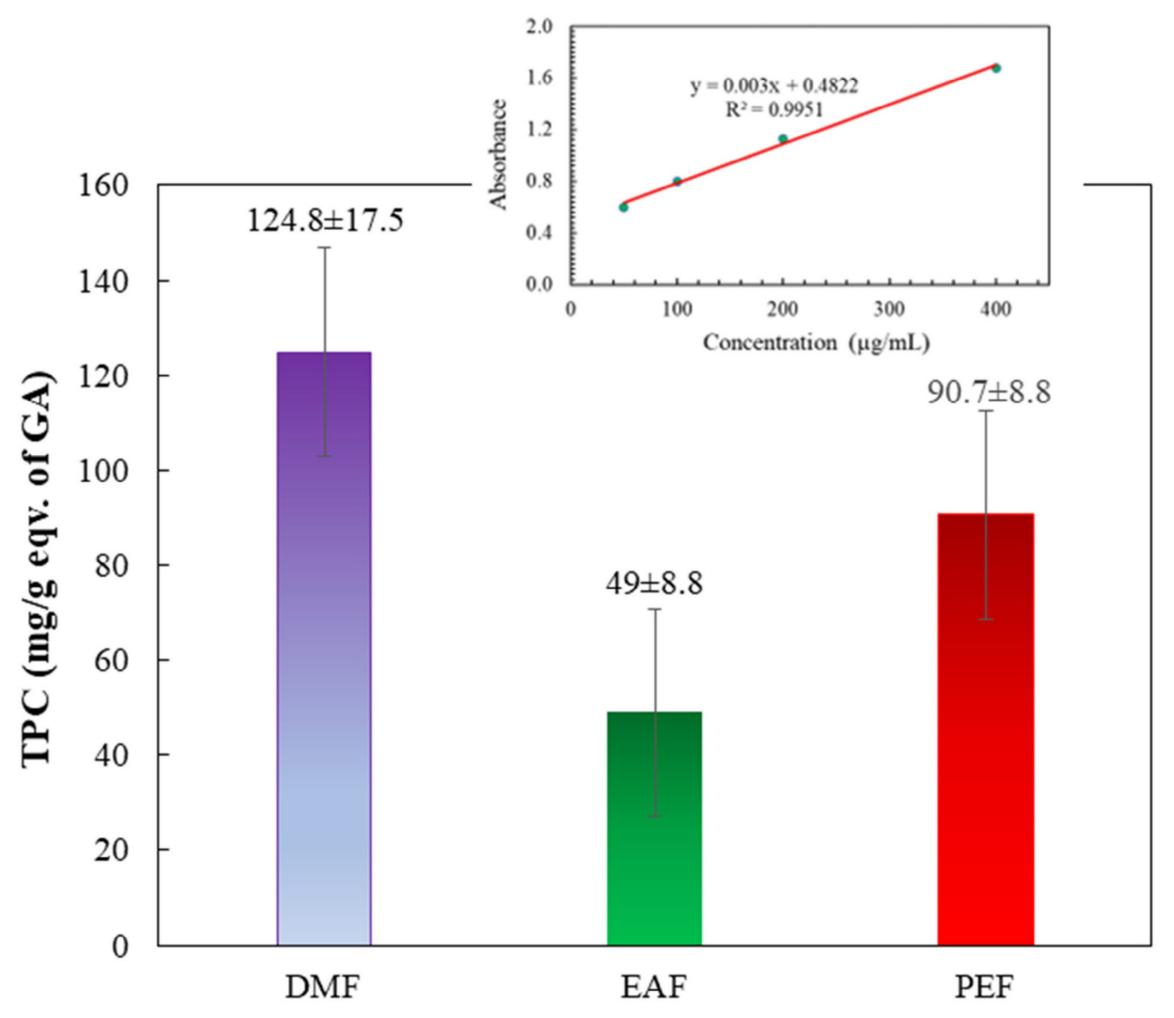
Total phenol contents of different fractions of *C. religiosa*. The inset shows the standard curve of gallic acid.

Figure 3 and its insert depict the total phenolic contents of the fractions. These contents were determined by applying the standard curve of GA (y = 0.003x + 0.4822; R² = 0.9951). The findings revealed that the DMF treatment showed the highest phenolic content, followed by the PEF treatment. Conversely, the EAF exhibited the lowest phenolic content. The phenol contents of the fractions followed this descending order: DMF > PEF > EAF.

### 3.4. Determination of total flavonoids

**Figure 4:**
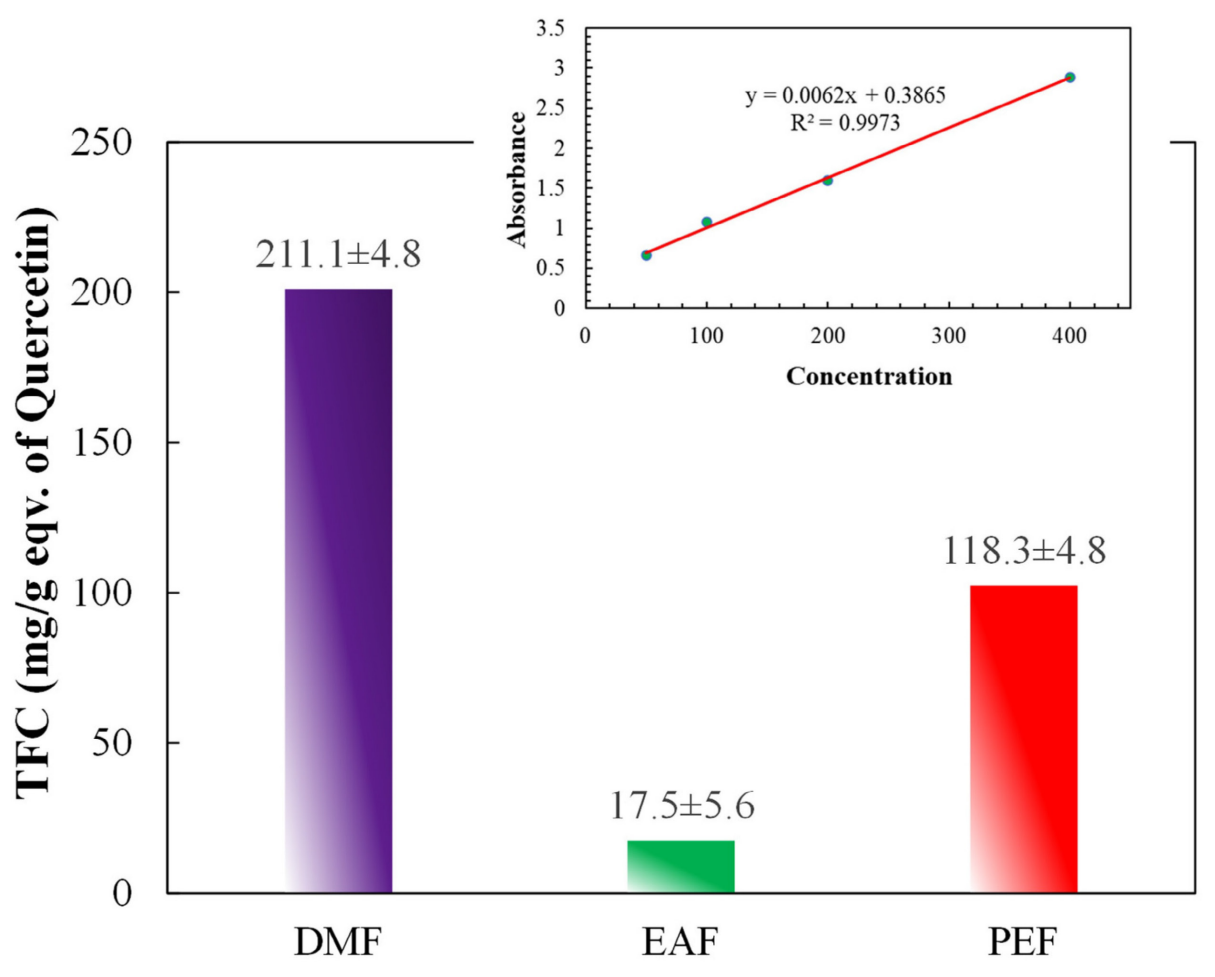
Total flavonoid contents of different fractions of *C. religiosa*. Inset shows the standard curve of quercetin.

The total flavonoid contents are demonstrated in Figure 4 and the inset of Figure 4. The contents were computed by employing the standard curve of quercetin (y = 0.0062x + 0.3895; R² = 0.9973) and were stated as QE per gm of plant extract. The outcomes revealed that the maximum flavonoid content was in the DMF fraction, then in the PEF, whereas the lowest content was found in the EAF. Thus, the flavonoid contents of the fractions decreased in the order of DMF > PEF > EAF.

### 3.5. *In vitro* AChE and BChE enzyme activity

**Figure 5:**
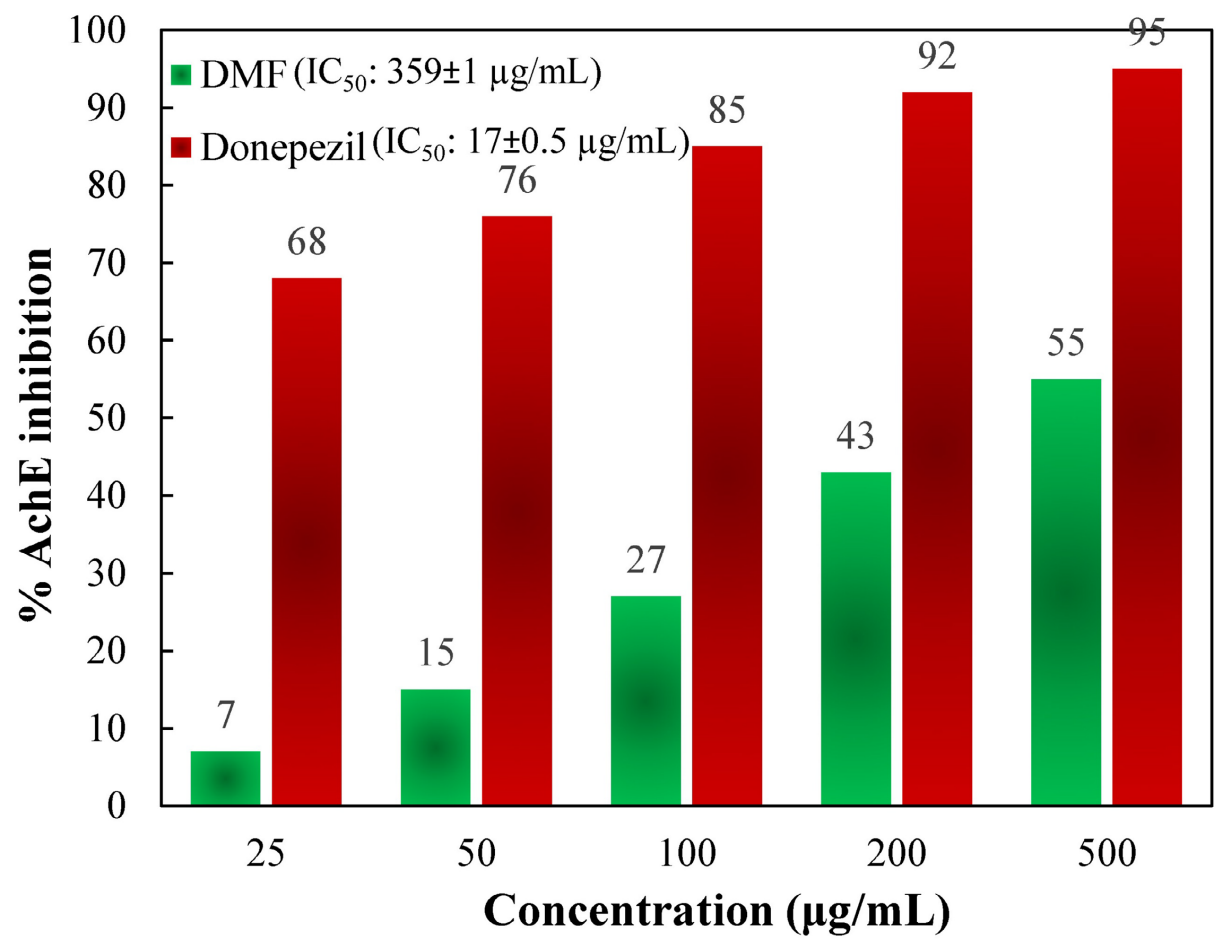
% inhibition of AChE activity at different concentrations of DMF and the reference standard donepezil.

**Figure 6:**
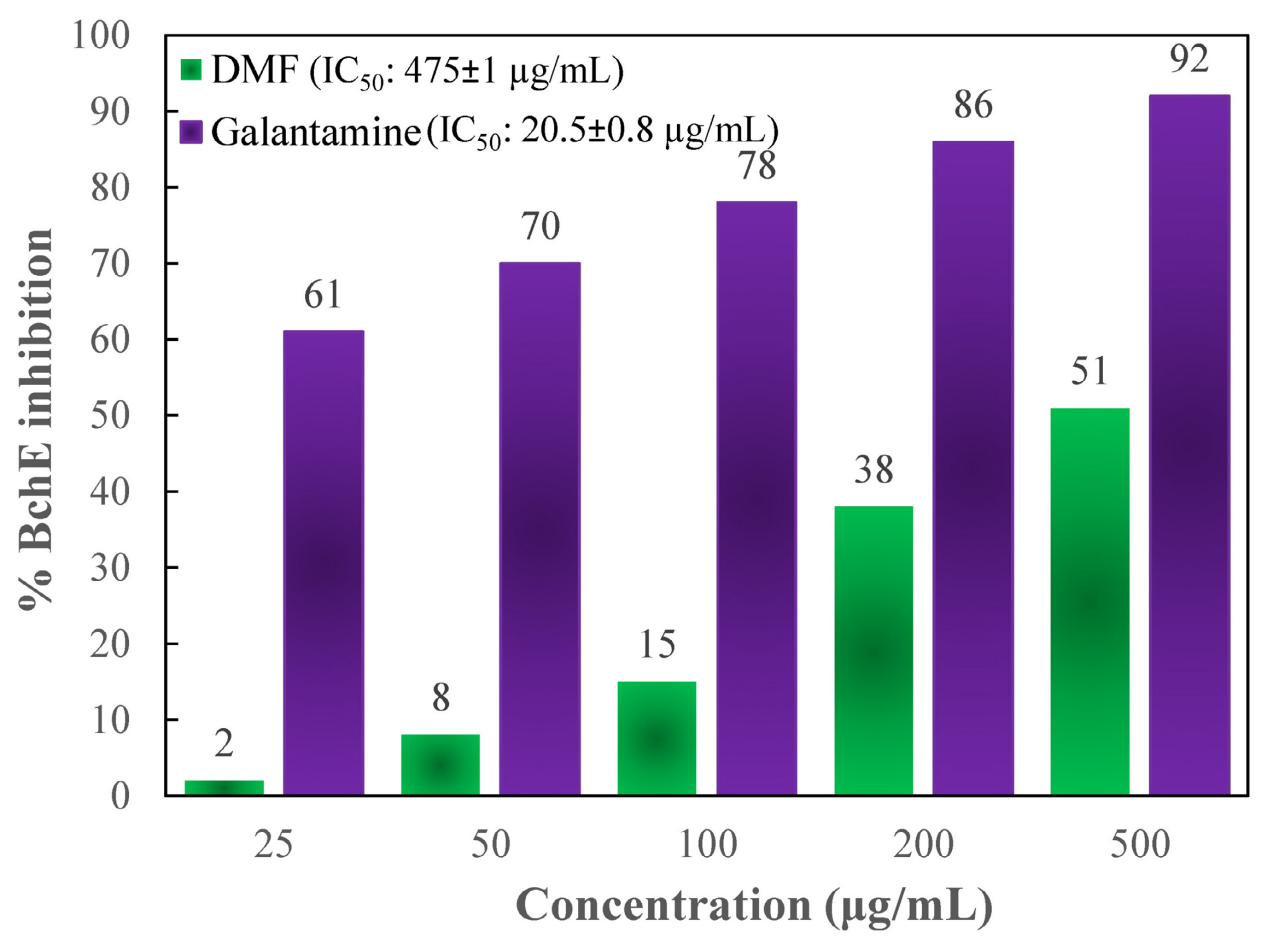
% inhibition of BChE at different concentrations of DMF and the reference standard galantamine.

To determine the activity of the DMF fraction as an anti-AD drug, its AChE as well as BChE inhibitory actions were measured. In comparison to those of the standards, Figures 5 and 6 depict the AChE and BChE inhibitory activities, respectively, and the cholinesterase inhibitory action showed dose-dependent changes. As demonstrated in Figure 5, the AChE inhibitory capacities of the DMF fraction were 7 ± 0.8, 15 ± 2.3, 27 ± 3.7, 43 ± 1.9, and 55 ± 2.2 % at concentrations of 25, 50, 100, 200 and 500 μg/mL, respectively, with an IC_50_ value of 359 ± 1 μg/mL. Besides, Figure 6 demonstrated the BChE inhibitory activities of DMF were 2 ± 1.3, 8 ± 0.6, 15 ± 1.5, 38 ± 1.9, and 51 ± 3.7 % at concentrations of 25, 50, 100, 200 and 500 μg/mL, respectively, with an IC_50_ value of 475 ± 1 μg/mL. In these experiments, donepezil and galantamine were applied as reference standards, and their IC_50_ values against AChE and BChE were 17 ± 0.5 and 20.5 ± 0.8 μg/mL, respectively.

### 3.6. Effects of different fractions on the MTT assay

**Figure 7:**
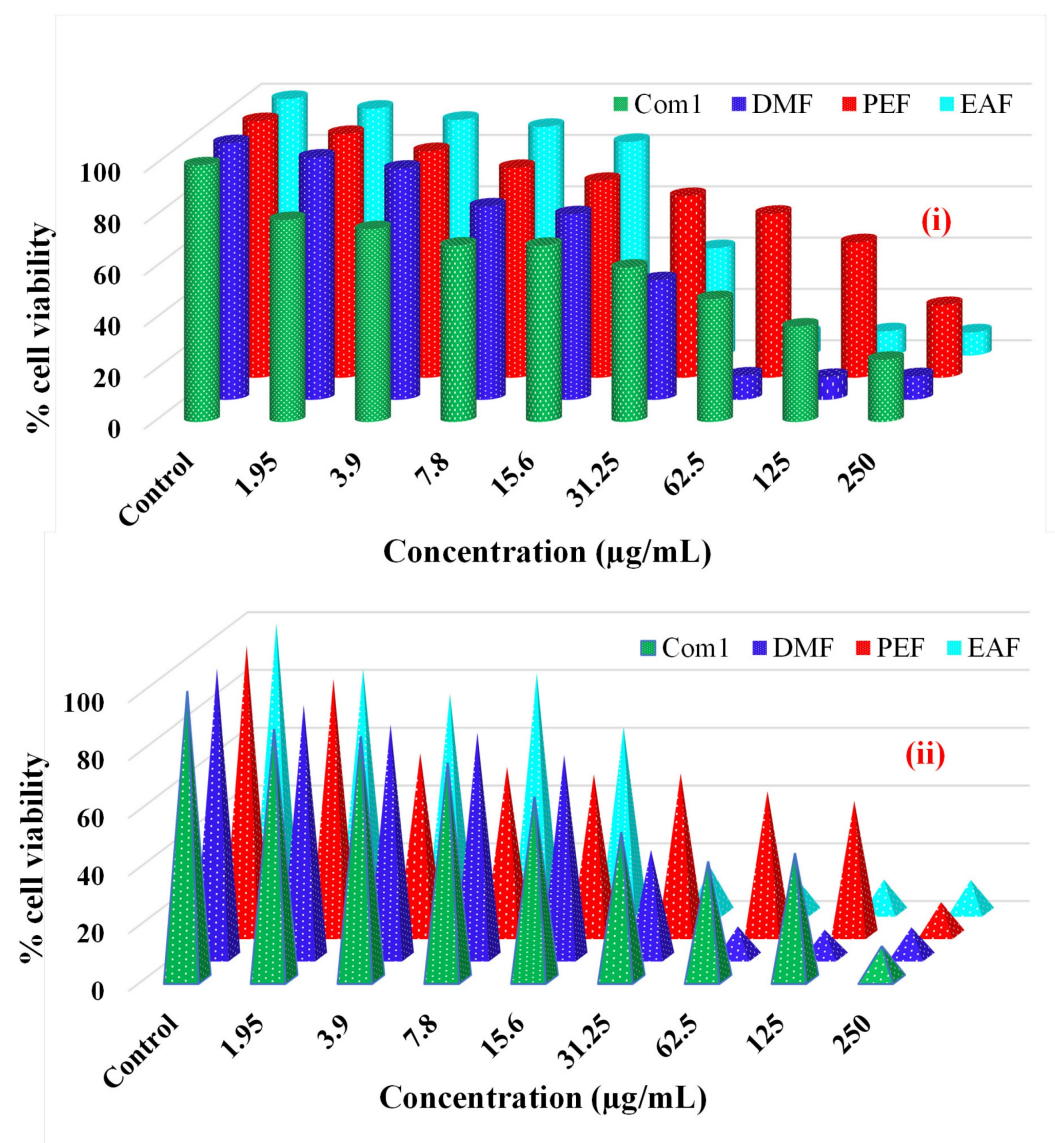
Effects of different concentrations of crude methanolic extract (Com1), DMF, PEF, EAF as % cell viability on (i) liver cancer cell line (ii) lung cancer cell line as measured by MTT 72 h following exposure.

The effectiveness of the crude methanolic extract (Com1) and three fractions resultant from the bark of *C. religiosa* in combating cancer was estimated by employing the MTT assay. The findings are presented as % of viable cells compared to the control after 72 hours of exposure, as demonstrated in Figure 7 (i and ii).

For the liver cancer (HepG2) cell line, Com1 exhibited cell viability % of 78.67, 75.09, 68.7, 68.61, 60.08, 47.84, 37.16, and 24.2 at doses of 1.95, 3.9, 7.8, 15.6, 31.25, 62.5, 125, and 250 µg/mL, respectively, compared with controls (0.5 % DMSO in a 5 % serum-containing DMEM medium). In contrast, the viability % in the presence of DMF were 94.4, 90.07, 75.35, 72.3, 46.57, 9.44, 8.88, and 9.16 across the same dosage range. Similarly, the % of viable cells for the PEF treatment were 94.98, 88.29, 81.73, 76.68, 70.78, 63.88, 52.79, and 28.27, while for the EAFs, they were 96.22, 91.88, 89.12, 83.27, 41.85, 8.77, 9.45, and 9.02. Finally, for this cell line, the DMF fraction showed the best efficacy.

In the case of the lung cancer (A549) cell line, Com1 demonstrated the viability of 86.93, 84.34, 75.23, 63.45, 51.13, 41.04, 43.92, and 11.62 % at doses mentioned earlier, compared with the controls. The presence of DMF resulted in the viability of 87.36, 80.67, 77.79, 70.04, 37.17, 10.68, 9.5, and 10.34 % across the same dosage range. The PEF treatment showed the viability of 88.48, 62.89, 58.15, 55.59, 55.87, 49.75, 46.35, and 11.28 %, while the EAF treatment displayed 84.11, 75.41, 82.66, 64.21, 15.44, 11.58, 11.29, and 11.17 %. Thus, EAF was the most active fraction against this cell line.

## 4. Discussion

Oxidative stress is basically determined by the uncontrolled synthesis of ROS and reactive nitrogen species (RNS). Oxidative stress originated through free radicals, for instance, superoxide anion radical (O_2_)^•-^, hydroxyl radical (•OH), H_2_O_2_, singlet oxygen (^1^O_2_), and peroxyl radicals (ROO^•^), which have great affinity for accepting an electron by combining with biological macromolecules, for example, lipids, proteins, and DNA. This affinity for oxidative stress leads to many human degenerative diseases 42. The antioxidative defence system works by removing ROS and preventing cellular destruction by neutralizing free radicals, thus defending against diseases like AD 43. However, severe biological destruction can happen when the rate of free radical synthesis is greater than the activity of the protection system, which increases the ROS level in the body 44. Increased levels of ROS perform an action in the pathogenesis of AD 45. In this study, as demonstrated in Figure 1, DMF demonstrated the highest % of DPPH radical scavenging, which reflects its potent antioxidant activity. The phosphomolybdenum assay also revealed similar findings, as demonstrated in Figure 2. Therefore, the scavenging potential of DMF was quantified via the use of DPPH and Phosphomolybdenum reagent to determine antioxidant activity 46. The degree of color variation in this assay is directly proportional to the concentration of antioxidants in the sample 47.

Further, phenolic compounds are very important alternatives to synthetic chemotherapeutic drugs and have been found to demonstrate effective antineoplastic effects against numerous steps of carcinogenesis and related inflammation. Phenolic and flavonoid compounds are the most vibrant classes of antioxidants that can scavenge free radicals to prevent cellular damage 48, 49. Figures 3 and 4 showed that the TPC and TFC for DMF are 124.8 ± 17.5 (mg/g eqv. of GA) and 211.1 ± 4.8 (mg/g eqv. of Quercetin), respectively, and high as compared to other fractions of *C. religiosa.* So, the phenolic and flavonoid contents increased with growing concentrations of the methanol extract, signifying that the bark of *C. religiosa* can reduce the threat of several degenerative diseases 50, including AD, by working as an antioxidant to prevent oxidative stress-induced cell damage.

Moreover, AD is a multifactorial chronic as well as a neurodegenerative syndrome that is diagnosed by the progressive deterioration of cognition, memory, and behavior; this disease typically has a very slow onset without rapid damage and ultimately leads to death. There are no available drugs that treat AD, but two hypothetical methods for the medication of AD have been advanced. The initial method works by quarantining prime progenitors to stop AD growth. The other mechanism includes symptomatic medication of the tertiary mental indications of AD 51. Currently, only a few cholinesterase inhibitors, for instance, donepezil, galantamine, and rivastigmine, as well as one fractional NMDA receptor antagonist, memantine, are FDA-permitted medicines for treating AD. Cholinesterase inhibitors are drugs that prevent the breakdown of acetylcholine inside synapses, resulting in a high level of acetylcholine. Therefore, researchers have tried discovering novel medicines from natural resources, like plants, that are highly effective at treating AD. In this present study, DMF was found to prevent AChE as well as BChE in a dose-dependent mode, as demonstrated in Figures 5 and 6, where the IC_50_ of DMF for AChE and BChE tests were 359 ± 1 μg/mL and 475 ± 1 μg/mL, respectively. The findings reveal that the high phenolic content in DMF not only enhances its antioxidant capacity but also suggests potential neuroprotective effects through cholinesterase inhibition. Studies have proven that the phenolic compounds in DMF can bind to the active sites of AChE and BChE, thereby inhibiting their activity. This dual action - antioxidant activity and cholinesterase inhibition - positions DMF as a promising candidate for further evaluation in AD research 52, 53.

Similarly, plant-derived products have been reported to be biologically safe for use with normal cells in the human body 3, 7. In this context, this was the first report in which EAF and DMF derived from the fresh bark of *C. religiosa* demonstrated potent antineoplastic activity. As demonstrated in Figure 7 (i) and (ii), this study was designed to govern the antiproliferative activity of these compounds against two selected types of cancer cells, A549 and HepG2, which represent lung adenocarcinoma and liver hepatocarcinoma, respectively, that are currently responsible for high mortality in humans 54, 55. In this study, healthy lung and hepatic cells were used as positive controls for these two cell lines. Our phytochemical fractions have shown selective efficacy on two types of cancer cells. For the HepG2 cell line, DMF was the most effective fraction, as the cell viability was the least among the fractions with IC_50_ 29.2 µM, and EAF was the best fraction worked against A549 cell lines, where the IC_50_ concentration was 24.7 µM.

These outcomes propose the significant action of the polyphenolic components of the methanolic extract in free radical neutralization and of DMF in the antiproliferative activity and inhibition of AChE action. Additional investigations are anticipated to identify the functioning compounds and leads that may be useful as candidate drugs.

## 5. Conclusion

The potent antioxidant activity and significant inhibition of AChE and BChE by the DMF fraction and the inhibition of cell growth by the EAF and DMF fractions of* C. religosa* focus on its prospect of affording an efficient treatment for AD and cancer, respectively. This work is the first effort to scrutinize the antioxidant, AChE/BChE inhibitory and antiproliferative activities of *C. religiosa*. It was found that the DMF fraction exhibited significant inhibition action against AChE and BChE. In the case of anti-proliferative activity, the DMF showed an IC50 value of 29.2 µM against the HepG2 cell line, while the EAF exhibited IC50 values of 24.7 µM in the A549 cell line. However, additional investigation in an animal model of AD and cancer is warranted to elucidate the *in vivo* efficacy and isolation of active metabolites of this plant.

## Supplementary Material

Supplementary figures and tables.
