# Supplementary material for: In vitro antioxidant, anticholinesterase, and antiproliferative activities of methanol extracts of Crateva religiosa bark
Source: J Cancer. 2025 Apr 28;16(8):2492–502. doi: 10.7150/jca.94492 (PMC12170497; doi:10.7150/jca.94492)
Supplement: Supplementary file 1 — Supplementary figures and tables. [file jcav16p2492s1.pdf]

In vitro antioxidant, anticholinesterase, and antiproliferative activities of methanol extracts of *Crateva religiosa* bark

Md Sifat Hossain[1, 2,ϒ], Md Abdul Majed Patwary[3,*,ϒ], Sharmin Akther Rupa[3], Mohsin Kazi[4], Ashok Kumar[5], Khalid M Alghamdi[5,*], Mohammed Mahbubul Matin[6], Md. Rezaur Rahman[7]

[1]*Department of Pharmacy, Comilla University, Cumilla-3506, Bangladesh*
[2]*Institute for Biomedical Science, Georgia State University, Atlanta, Georgia*
[3]*Department of Chemistry, Comilla University, Cumilla-3506, Bangladesh*
[4]*Department of Pharmaceutics, College of Pharmacy, PO BOX 2457, King Saud University, Riyadh, 11451, Saudi Arabia*
[5]*Department of Dermatology, College of Medicine, King Saud University, Riyadh-11451, KSA*
[6]*Department of Chemistry, University of Chittagong, Chittagong, 4331, Bangladesh*
[7]*Department of Chemical Engineering and Energy Sustainability, Faculty of Engineering, University Malaysia Sarawak*

***Corresponding author:** (M.A.M.P.) mampatwary@cou.ac.bd, (K.A.) kmgderm@gmail.com

[ϒ]These authors contributed equally to this work.

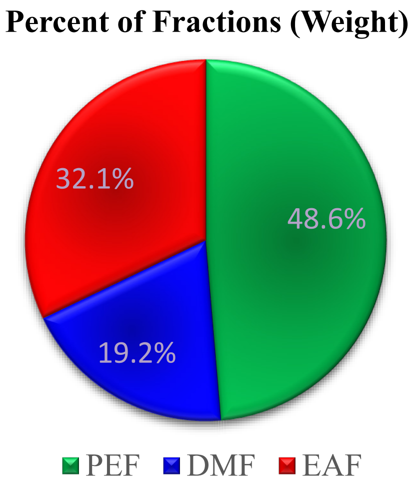

Figure S1: Percent of fractions (weight) of PEF, DMF, and EAF

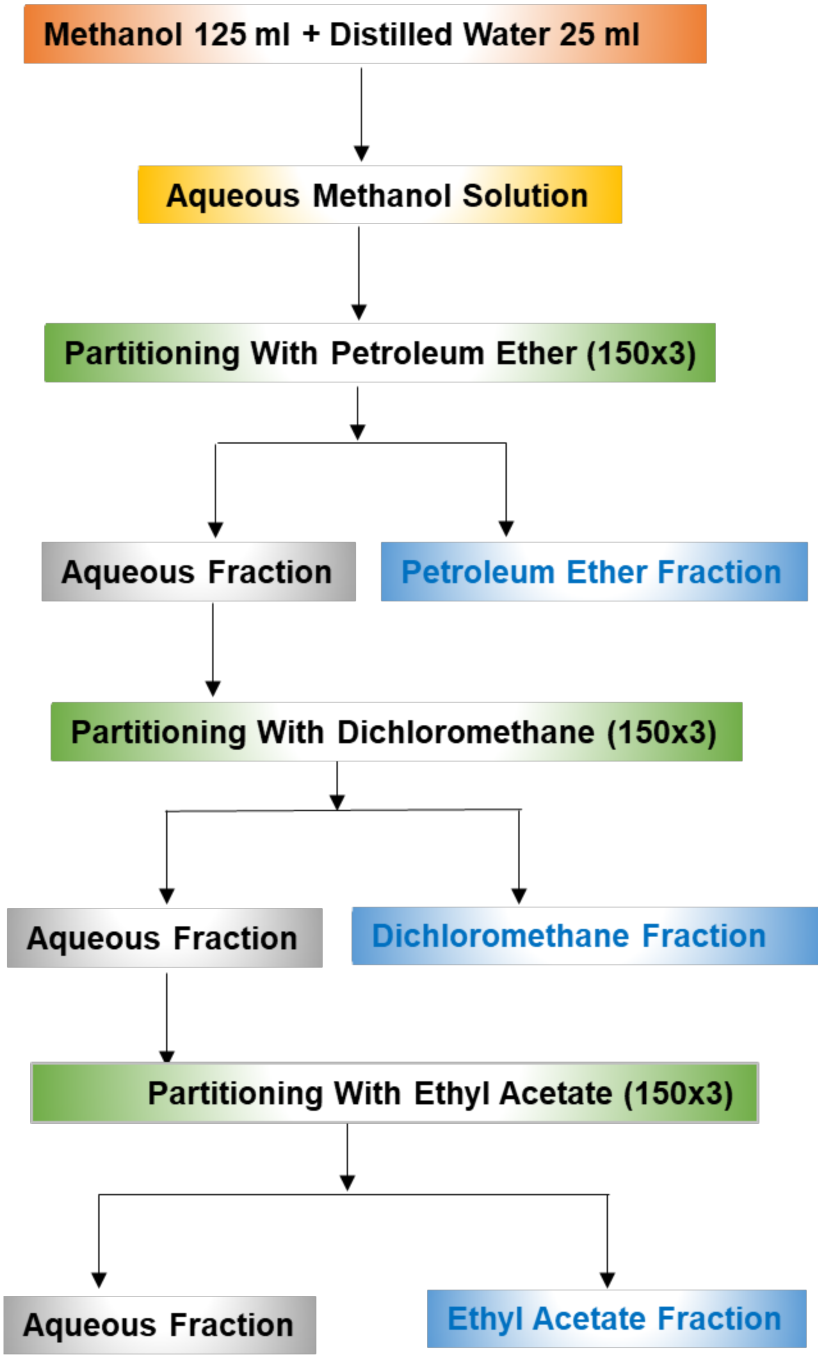

Flow Chart S1: Schematic representation of solvent-solvent partitioning of the crude methanol extract of *C. religiosa.*

Table S1: Significance values obtained from one-way ANOVA assessment of the means of three cell culture experiments examining the cytotoxicity of human liver cancer cell line treated with different drugs.

Concentrations of drugs (µg per mL)	p-value of drugs on human hepatocellular carcinoma			
	Com 1	DMF	PEF	EAF
Control vs 1.95	P < 0.0001	No	No	No
Control vs 3.9	P < 0.0001	No	P < 0.0001	No
Control vs 7.8	P < 0.0001	P < 0.0001	P < 0.0001	No
Control vs 15.6	P < 0.0001	P < 0.0001	P < 0.0001	No
Control vs 31.25	P < 0.0001	P < 0.0001	P < 0.0001	P < 0.0001
Control vs 62.5	P < 0.0001	P < 0.0001	P < 0.0001	P < 0.0001
Control vs 125	P < 0.0001	P < 0.0001	P < 0.0001	P < 0.0001
Control vs 250	P < 0.0001	P < 0.0001	P < 0.0001	P < 0.0001

Table S2: Significance values obtained from one-way ANOVA assessment of the means of three cell culture experiments examining the cytotoxicity of human lung cancer cell line treated with different drugs.

Concentrations of drugs (µg per mL)	p-value of drugs on adenocarcinomic human alveolar basal epithelial cells			
	Com 1	DMF	PEF	EAF
Control vs 1.95	P < 0.01	P < 0.01	No	No
Control vs 3.9	P < 0.001	P < 0.0001	P < 0.0001	P < 0.0001
Control vs 7.8	P < 0.0001	P < 0.0001	P < 0.0001	P < 0.01
Control vs 15.6	P < 0.0001	P < 0.0001	P < 0.0001	P < 0.0001
Control vs 31.25	P < 0.0001	P < 0.0001	P < 0.0001	P < 0.0001
Control vs 62.5	P < 0.0001	P < 0.0001	P < 0.0001	P < 0.0001
Control vs 125	P < 0.0001	P < 0.0001	P < 0.0001	P < 0.0001
Control vs 250	P < 0.0001	P < 0.0001	P < 0.0001	P < 0.0001

The statistical data were obtained for human lung cancer cell lines with different drugs with their different concentrations and for controls (no drug) using one-way ANOVA and the post hoc Tukey HSD test.
